# Hyaluronic Acid Combined with Ozone in Dental Practice

**DOI:** 10.3390/biomedicines12112522

**Published:** 2024-11-04

**Authors:** Alessio Rosa, Alberto Maria Pujia, Claudio Arcuri

**Affiliations:** 1 Department of Chemical Science and Technologies, Dentistry, University of Rome Tor Vergata, 00133 Rome, Italy; 2 Department of Biomedicine and Prevention, University of Rome Tor Vergata, 00133 Rome, Italy; 3 Department of Clinical Sciences and Translational Medicine, University of Rome Tor Vergata, 00133 Rome, Italy

**Keywords:** hyaluronic acid, ozone therapy, biocompatible material

## Abstract

Background: Topical applications have long been regarded as precise methods for delivering drugs to soft tissues, such as the periodontal ligament, as well as hard structures, including the alveolar bone and cementum. Recently, the combination of hyaluronic acid (HA) and ozone therapy has gained popularity as a supportive treatment for chronic inflammation, in addition to its established role in enhancing healing after dental procedures. Methods: To gather the necessary research for our study, we conducted a systematic search across several databases, including PubMed, Google Scholar, and Ovid. Our study was registered under Prospero number CRD42024569641. The research, which began in June 2015 and concluded in May 2024, systematically examined the potential benefits of topical HA application in the management of both acute and chronic inflammatory diseases. Using relevant keywords and Medical Subject Headings, we selected 28 pertinent studies: three related to gingivitis, thirteen on chronic periodontitis, seven involving dental surgeries such as implants and sinus lifts, and three on oral ulcers. Results: The risk of bias among the analyzed studies was assessed using the RoB 2 tool. Regarding the randomization process, 75% of the studies exhibited a high risk of bias. However, all studies (100%) had a low risk of bias concerning allocation concealment. Only 25% of the studies adequately addressed performance bias, and another 25% reported all outcome data. Nevertheless, 85% of the included studies showed a low risk of reporting bias. Conclusions: The results indicate that the use of topical HA in combination with ozone therapy is highly effective not only in promoting post-operative healing following dental procedures but also in providing positive outcomes for individuals dealing with chronic gingivitis, periodontal inflammation, and oral ulcers.

## 1. Introduction

Hyaluronic acid (HA), a naturally occurring glycosaminoglycan, does not contain sulfur and has a high molecular weight that ranges between 4000 and 20,000,000 Daltons [1]. The polymer structure of HA consists of polyanionic disaccharide units made of glucuronic acid and N-acetyl glucosamine, linked together by alternating β-1,3 and β-1,4 glycosidic bonds. HA is present throughout the body, especially in the extracellular matrix of connective tissues, where it plays an essential role. It can be found in synovial fluid, embryonic mesenchyme, vitreous humor, skin, and more [2,3]. In dentistry, HA is crucial for maintaining the health of soft periodontal tissues, such as the gingiva and periodontal ligament, as well as hard tissues like alveolar bone and cementum, contributing to various physiological functions.

One of HA’s key roles is regulating the inflammatory response. In periodontal tissues like the gingiva, periodontal ligament, and alveolar bone, HA is produced in high-molecular-weight forms by hyaluronan synthase enzymes. However, during chronic inflammation, such as gingivitis or post-operative recovery after implants or sinus lift surgery, this high-molecular-weight HA is broken down into smaller molecules [4,5]. This degradation is accelerated by reactive oxygen species (ROS), including superoxide and hydroxyl radicals, produced by inflammatory cells such as polymorphonuclear leukocytes during bacterial phagocytosis in periodontal disease. These smaller HA fragments signal tissue damage and trigger the recruitment of immune cells to the injury or infection site. In contrast, intact high-molecular-weight HA helps modulate the immune response, preventing excessive inflammation [5,6]. Furthermore, HA is an integral part of the sequential steps of the wound-healing process, which includes inflammation, granulation tissue formation, epithelialization, and tissue remodeling in both mineralized and non-mineralized tissues [7]. The broad spectrum of functions attributed to HA has stimulated advances in the development and application of HA-based biomaterials for the treatment of various inflammatory conditions. Given the multifunctional role of HA in wound healing and the similarity of biological principles governing gingival and bone healing, it is plausible that HA exerts comparable beneficial effects in the healing processes of mineralized and non-mineralized periodontal tissues [8]. The use of HA spans several branches of medicine and its safety profile is further exemplified by the absence of contraindications or drug interactions [9,10].

HA is widely used across different medical fields due to its safety, with no reported contraindications or drug interactions. In recent years, topical HA formulations have been developed to aid in the treatment of both acute and chronic oral conditions, such as post-surgical healing, supported by numerous animal studies [11]. While there have been reviews on HA’s role in treating periodontal diseases, a comprehensive assessment of its therapeutic benefits in managing both acute and chronic inflammatory oral diseases is still lacking. Our study aims to systematically review the available literature on HA’s therapeutic effects, categorize its main dental applications, clarify its pathophysiological basis, outline post-operative application protocols, and identify the most effective usage parameters for HA in dentistry [12,13,14].

Additionally, ozone therapy has gained recognition as a powerful adjunct treatment in dentistry due to its antimicrobial, anti-inflammatory, and healing-promoting properties [15,16,17]. When combined with HA, ozone therapy offers a synergistic effect, enhancing treatment outcomes in various dental conditions. Ozone destroys microorganisms by damaging their cell membranes through ozonolysis and causes intracellular changes via secondary oxidative effects, leading to protein oxidation and loss of organelle function. It acts as a potent disinfectant without producing toxic byproducts, selectively targeting microbial cells without harming healthy ones. Ozone halts all vital bacterial functions within seconds of application, with Gram-positive bacteria being more susceptible than Gram-negative ones. In dental care, ozone disrupts bacterial cells, helping eliminate acidogenic bacteria that cause dental caries [17].

The combination of ozone therapy and HA has been successful in wound healing, oral lichen planus, gingivitis, periodontitis, halitosis, osteonecrosis of the jaw, post-surgical pain, plaque and biofilms, root canals, dentin hypersensitivity, temporomandibular joint disorders, and teeth whitening. This systematic review seeks to explore the combined use of HA and ozone in dentistry, emphasizing their roles in managing various dental conditions and procedures.

## 2. Materials and Methods

To conduct the research for our study, we performed a systematic search across various databases, including PubMed, Google Scholar, and Ovid. The study was registered under Prospero with the identifier CRD42024569644. Starting in June 2015, we carried out a systematic review focusing on the potential benefits of topical hyaluronic acid (HA) application in managing both acute and chronic inflammatory conditions. After an initial screening of 278 articles, 78 were fully reviewed, and only 28 were ultimately selected for inclusion in the analysis. The PICO question guiding the study was: “In randomized controlled clinical trials, is the combination of HA and ozone effective in promoting oral health by treating gingivitis, ulcers, wounds, and gingival recession compared to control groups?” (Table 1 and Table 2).

We utilized a combination of keywords and, for PubMed, specific Medical Subject Headings (MeSH) to refine our search strategy. These included terms like “hyaluronic acid and ozone and periodontitis”, “hyaluronic acid and ozone and gingivitis”, and other similar phrases relevant to dental conditions and treatments involving HA and ozone. No restrictions were placed on publication year. Our literature selection process strictly followed PRISMA guidelines, and we established clear inclusion criteria: studies had to be published in English, involve human-controlled trials, and report either histological or clinical evaluations of hyaluronic acid’s impact in dental disease contexts. We excluded document types that did not represent primary research, such as literature reviews, technical notes, letters to editors, and instructional courses.

**Figure 1:**
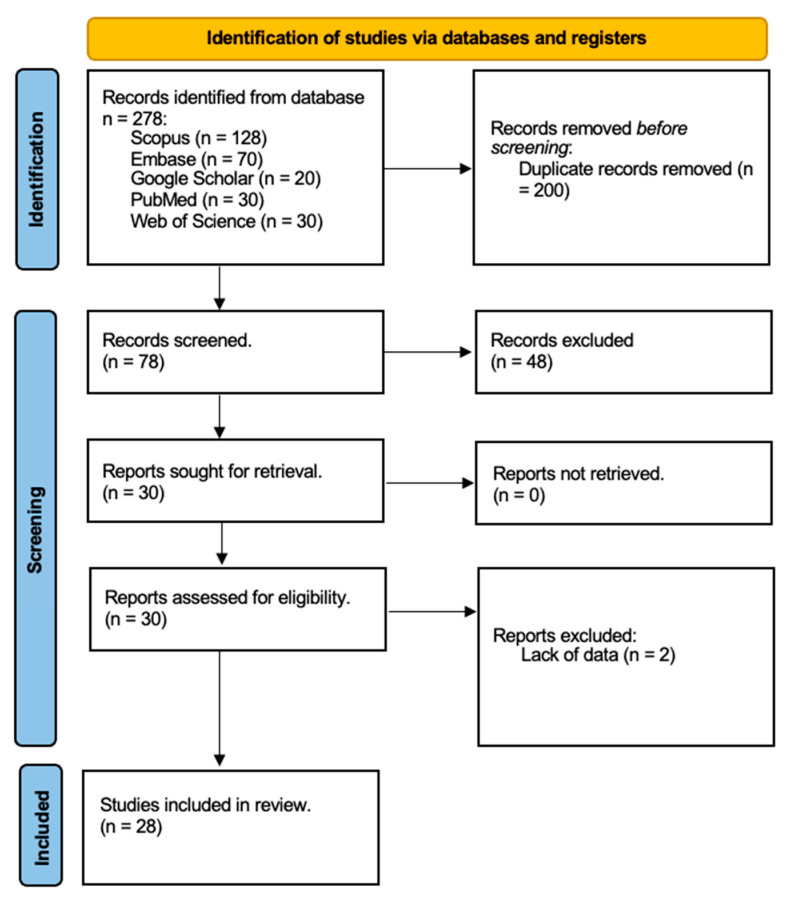
Search strategy flow chart of studies selected from databases (Scopus, Embase, Google Scholar, PubMed, Web of Science). Reports excluded for lack of data.

Two authors, A.R. and A.M.P., independently reviewed the full texts of selected articles to assess their relevance to the topic, excluding any that did not meet the criteria for analysis. Additionally, we examined the reference lists of the remaining articles to identify any relevant studies that might have been missed during the electronic search. Duplicate entries were removed, and we focused on studies that directly addressed our topic of interest. Animal studies and in vitro research were excluded, as we concentrated exclusively on human trials. We also eliminated studies that explored the use of hyaluronic acid in treating. After completing our meticulous selection process, we concluded with a final tally of 28 relevant publications for our review, as depicted in Figure 1 of our study documentation.

## 3. Results

Navigating through the world of dentistry, the role of hyaluronic acid (HA) emerges not just as a treatment modality but as a beacon of innovation, bridging traditional practices with the promise of enhanced healing and patient comfort. The journey into its application spans various facets of dental care, each illuminated by studies that not only underscore its efficacy but also hint at the broader potential of HA in revolutionizing dental treatments (Table 3).

### 3.1. Quality Assessment and Risk of Bias

**Figure 2:**
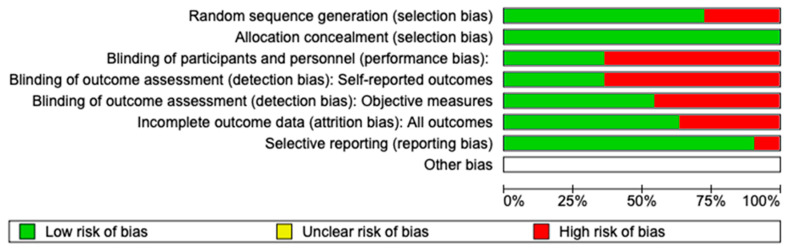
Figure shows a graph representing the risks of bias in various aspects of an analysis. The risks are coded in three colors: green for “low risk of bias”, and red for “high risk of bias”. In general, most categories show a low risk of bias (in green), except for the participant and staff blinding and the outcome assessment blinding, where there is a significant percentage of a high risk of bias (in red).

**Figure 3:**
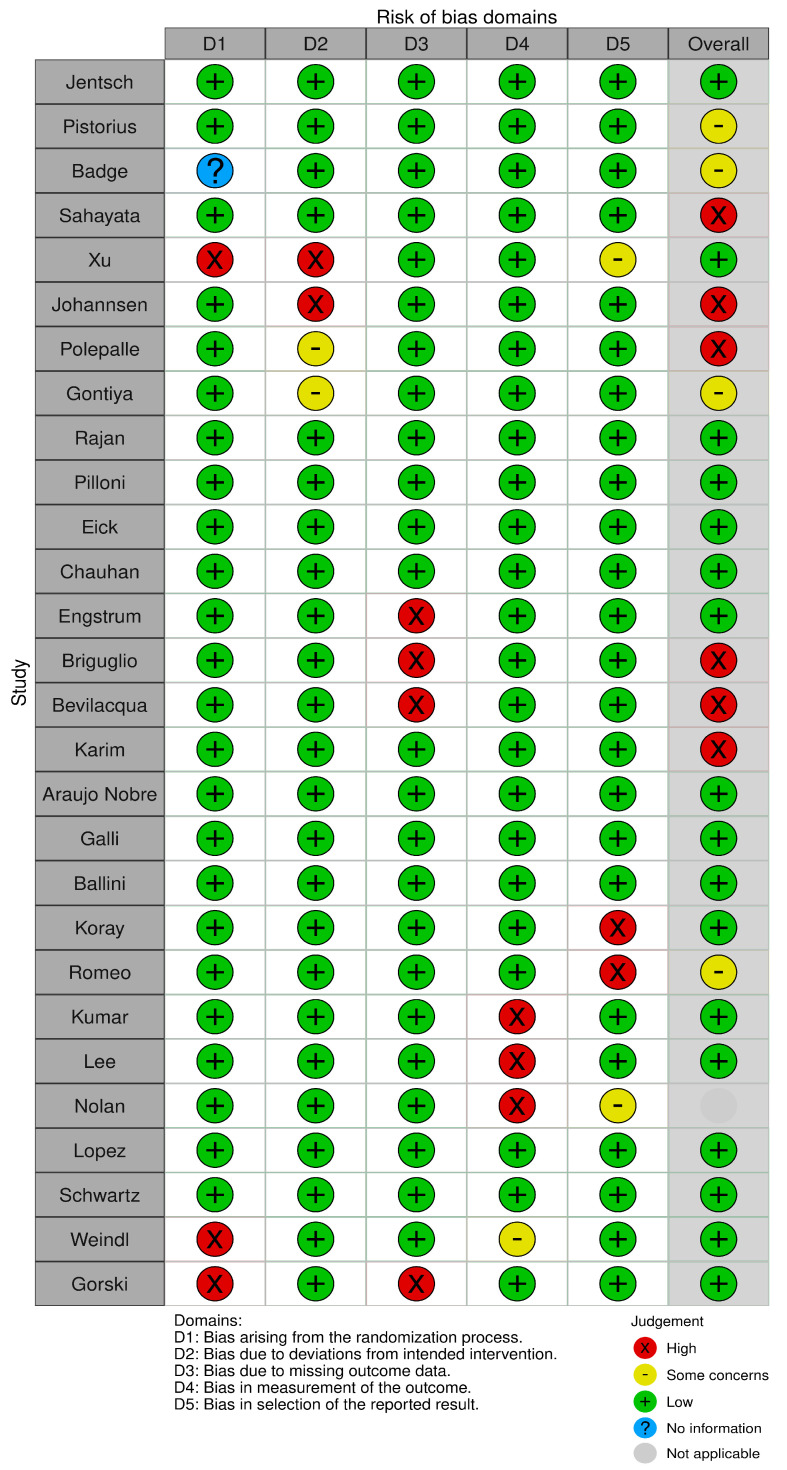
Risk of bias. The image shows a table assessing the risk of bias in different categories (D1–D5) for various studies. The categories include bias from the randomization process, deviations from the intended intervention, missing outcome data, outcome measurement, and selection of reported outcomes. The judgments are coded with symbols: green (+) indicates low risk of bias, yellow (−) represents some concerns, red (×) indicates high risk, blue (?) indicates no information, and gray shows non-applicability.

Two reviewers, A.M.P. and A.R., evaluated the risk of bias using the Cochrane Risk-of-Bias Tool for randomized trials (RoB 2). In cases of disagreement, a third reviewer, C.A., was consulted to reach a consensus (Figure 2). The estimated risk of bias for the studies analyzed, based on RoB 2, is displayed in Figure 2 and Figure 3. For the randomization process, 75% of the studies were found to have a high risk of bias. In terms of allocation concealment, all studies (100%) had a low risk of bias. However, only 25% of studies successfully avoided performance bias, and 25% reported all outcome data. Notably, 85% of the studies included in the review were deemed to have a low risk of reporting bias (Figure 3).

### 3.2. In the Battle Against Gingivitis

The tale of HA combined with OT begins in the realm of gingivitis, where its prowess was put to the test. The study by Jentsch et al. [2] turns the spotlight on how a seemingly simple regimen of applying 0.2% HA topically twice daily can lead to remarkable improvements in oral health markers such as plaque indices and papillary bleeding index (PBI), almost heralding a new dawn in non-invasive gingivitis management. The test group showed significant improvement in the study area for the plaque indices beginning with day 4 (*p* = 0.011) and the PBI beginning with day 7 (*p* = 0.001) in comparison with the placebo group. The crevicular fluid variables were significantly improved in the center of the studied inflammation area in the test group. This narrative is further enriched by Pistorius et al. [16], who, through their exploration of an HA spray, reveal its potent effect in curbing sulcus bleeding. It is a testament to the versatility of HA, showing that whether in gel or spray form, its therapeutic potential remains undiminished.

Sahayata [17] et al.’s work adds depth to this story, highlighting how HA and OT, when wielded alongside traditional scaling and oral hygiene practices, can significantly outshine placebo treatments. It is as if HA whispers to the inflamed gingiva, coaxing it back to health more effectively than conventional methods alone. Clinically, there is significant difference (*p* < 0.05) for GI and PBI in test group as compared to other groups, but reduction in PI was non-significant. In negative control and placebo control groups, the difference between clinical parameters was non-significant.

### 3.3. Chronic Periodontitis: A New Frontier

The narrative then shifts to the challenging terrains of chronic periodontitis, where HA’s role expands from a supportive actor to a protagonist [19]. The local application of HA-OT gel emerges not just as a treatment but as a beacon of hope, reducing proliferation indices and quelling the inflammatory onslaught, thereby charting a new course in periodontal healing. Jentsch [2] showed that the subgingival application of 0.2% hyaluronic acid gel (GENGIGEL^®^) with SRP in chronic periodontitis patients improved the GI and bleeding index (BI) when compared with control sites, as confirmed by a gingival biopsy, which showed a significant reduction of inflammatory infiltrate.

The saga deepens with the collaborative efforts of Johannsen et al. and Polepalle et al. [14], who, through their meticulous research, unveil the symbiotic potential of HA and scaling and root planing (SRP). Their findings sing praises of HA’s ability to significantly reduce bleeding, improve clinical attachment levels, and even alter the microbial landscape, painting a picture of a future where HA could stand as a cornerstone of periodontal therapy [20]. Subgingival administration of 1 mL 0.2 mL 0.8% HA gel once a week for 6 weeks ameliorated the sulcus fluid flow rate (SFFR).

### 3.4. Surgical Frontiers and Implant Surgery

The versatility of HA with OT transcends the non-surgical domain, stepping boldly into surgical arenas. Here, the work of Araújo Nobre [30] et al. shines a light on HA’s role in enhancing the healing milieu of the peri-implant complex, offering a glimpse into its potential to improve implant success rates [22]. Statistically significant differences were found in favor of the HA group in the modified bleeding index on the second observation (*p* = 0.003). The difference was more marked in the axial implants placed in the fifth sextant (*p* = 0.05). The correlation coefficient between plaque and bleeding index revealed a potentially better result for CHX at 6 months.

Bagde et al.’s [18] exploration of HA and OT in treating deep periodontal defects not only highlights their efficacy in reducing pocket depth but also subtly hints at their role in regenerative dentistry. Meanwhile, Ballini et al.’s [32] research suggests HA’s promise in bone regeneration, providing a beacon of hope for those facing the daunting prospects of bone loss. Recession depths in the first, third, and sixth month were 1.82 ± 0.442, 1.31 ± 0.47 mm, and 0.91 ± 0.29, respectively, which showed a significant reduction from the baseline. Recession widths in the first, second, and third weeks were 3.04 ± 0.442 mm, 1.31 ± 0.47 mm, and 1.49 ± 0.59 mm, respectively. There was a statistically significant reduction (*p* > 0.005) [18].

### 3.5. The Healing Touch on Oral Ulcers

In the realm of oral ulcers, HA emerges as a gentle healer. Nolan [6] and Lee et al.’s [35] investigations into its efficacy in treating recurrent aphthous ulcers and Behçet’s disease not only underscore its therapeutic potential but also offer comfort to sufferers, promising a future where pain and discomfort are but distant memories [36,37]. A subjective reduction in the number of ulcers was observed in 72.7% of the patients. A decrease in the ulcer healing period was observed in 72.7% of the patients; 75.8% experienced improvement in VAS for pain.

Through this detailed narrative, hyaluronic acid emerges not merely as a molecule but as a harbinger of a new era in dentistry [7,23,25,26,27,39]. Each study, with each finding, adds a layer to our understanding, painting a picture of a future where HA stands as a pillar of dental care, bridging the gap between traditional methods and the promise of regenerative, minimally invasive treatments [31,34,38]. It is a story of transformation, of hope, and of the relentless pursuit of betterment in dental care, heralding a future where patient comfort and healing are paramount [28,29,33].

## 4. Discussion

The discussion on hyaluronan (HA) highlights its therapeutic potential in dentistry, particularly for wound healing and periodontal health. However, to strengthen future research, it is essential to include specific statistical analyses, elucidate the mechanisms by which HA promotes healing, and define clear patient selection criteria. Statistical measures should encompass quantitative outcomes like reductions in gingival inflammation and pocket depths, alongside details on the statistical methods used, such as *t*-tests and ANOVA, to analyze data effectively. The mechanisms through which HA aids wound healing involve hydration, promotion of cell migration and proliferation, anti-inflammatory effects, stimulation of angiogenesis, interaction with the extracellular matrix (ECM), and biochemical signaling through cell surface receptors. Clear patient selection criteria are crucial, including age limits, specific diagnoses, and severity levels, while excluding those with confounding comorbid conditions, relevant medications, or allergies to HA. Future studies should also prioritize randomization and blinding to enhance validity. The referenced study by Boccalari et al. exemplifies how well-structured clinical trials can yield valuable insights into HA’s effects in dental procedures, underscoring the need for high-quality research to optimize HA’s use in clinical dental practices and improve treatment protocols for periodontal and oral health conditions.

Hyaluronan, a versatile glycosaminoglycan integral to the extracellular matrix in vertebrate tissues, is renowned for its pivotal role in scar-free wound healing and has significant implications in oral health and dentistry. Exploring the literature reveals compelling evidence positioning hyaluronan at the core of periodontal tissue healing, highlighting its promising application in managing periodontal diseases. HA has proven to be a valuable clinical tool across fields such as ophthalmology, osteology, and dermatology due to its unique biochemical and biophysical properties. In dentistry, HA-based products have shown effectiveness in managing gingivitis through both anti-inflammatory and antiedematous effects. Studies have demonstrated that HA gels, particularly when used alongside mechanical treatments like scaling, significantly reduce gingival inflammation. However, the overall efficacy of HA in periodontal therapy shows variation, attributed to different product formulations, application methods, and study biases, making it challenging to recommend a specific approach [34,40].

Research into the use of HA in treating chronic periodontitis has shown improvements in gingival health when combined with scaling and root planing, although the impact on deeper periodontal parameters is less pronounced. Other studies have explored HA’s role in surgical periodontal therapy and bone regeneration, with positive outcomes in bone growth when used with autologous bone or as a filler in bone cysts. The application of HA in managing temporomandibular joint disorders (TMJDs) and oral ulcers, including those from Behçet’s disease, has also been reported, showcasing its potential for pain reduction and healing enhancement. Despite these advancements, the exact mechanisms by which HA influences cell behavior and tissue regeneration remain unclear, highlighting the need for further research [21,41].

Following gingivectomy surgery, wounds heal through secondary intention, leading to discomfort and slower recovery compared to primary intention healing. To accelerate healing and alleviate discomfort, photobiomodulation (PBM) has emerged as a promising supplementary treatment. Studies have consistently demonstrated that PBM therapy is an effective method to enhance recovery after a gingivectomy [42]. Additionally, various topical agents have been shown to aid in post-gingivectomy wound healing, including HA gel, herbal gels, applications of non-thermal atmospheric pressure plasma, and Vitrocure^®^ gel. Furthermore, research by Turgut Çankaya [43] and colleagues evaluated the effect of HA application following laser-assisted frenectomy, concluding that HA was a viable option for reducing the wound surface area within 14 days and serving as a wound dressing post-frenectomy. The soft-tissue-healing potential of HA, as observed in these studies, may be explained by its anti-inflammatory properties and its ability to induce epithelial tissue formation and increase connective tissue vascular supply histologically [44].

Combining hyaluronan with ozone therapy has shown potential in enhancing the therapeutic effects of HA. Ozone, known for its antimicrobial and healing properties, can complement HA’s benefits, offering a dual approach to managing oral health conditions. The combination of HA and ozone has been explored in various studies, indicating promising results in accelerating wound healing, reducing inflammation, and improving periodontal health. This synergistic approach harnesses the strengths of both agents, potentially leading to more effective treatments for gingivitis, periodontitis, and post-surgical recovery [45,46].

The future of HA and ozone therapy in clinical settings looks promising, aligning with the goals of translational and evidence-based medicine, paving the way for personalized treatment approaches. Hyaluronan’s benefits extend far beyond the superficial layers of the marginal gingiva, reaching into the depths of periodontal tissues. It leverages its well-documented wound-healing mechanisms to alleviate discomfort and accelerate healing, particularly beneficial in the context of gingivitis and chronic periodontitis. Moreover, HA’s potential shines in the aftermath of surgical interventions, such as implants and sinus lifts, where its topical application, combined with ozone therapy, can significantly hasten the healing process [29]. This not only translates to quicker recovery times but also considerably mitigates post-surgical discomfort, making the healing journey smoother and more bearable [21,23,24,25,26,27,28,29,31,32,33,34,35,36,37,38,39,40,41,42,43,44,45,46,47].

In the battle against oral ulcers, the combination of hyaluronan and ozone therapy emerges as a formidable ally, showcasing its therapeutic prowess in a comprehensive dental care strategy. The localized approach of topical treatments ensures high concentrations of therapeutic agents are delivered directly to the teeth and oral mucosa, enhancing effectiveness compared to systemic routes. The path forward beckons for more granular research, particularly laboratory investigations and large-scale, randomized controlled clinical trials. These future studies hold the key to unlocking the full potential of hyaluronan and ozone therapy as carriers for periodontal tissue cells, potentially revolutionizing tissue regeneration techniques for both mineralized and non-mineralized periodontal tissues.

Questions linger regarding the optimal administration modalities—be it through sprays, gels, or nebulization—and the most effective post-operative treatment schedules tailored to each dental condition. These inquiries pave the way for a deeper exploration of the combined role of hyaluronan and ozone therapy in dentistry, hinting at a future where their application is as nuanced as it is transformative. As we peer toward the horizon, the promise of hyaluronan and ozone therapy in enhancing dental care and patient healing journeys is undeniable. Their journey from components of the extracellular matrix to cornerstones of dental therapeutics is a testament to the power of harnessing nature’s healing mechanisms, offering a brighter, pain-free future for patients worldwide [48].

Our research has several important limitations: first, we only included studies published in English. While this ensured consistency in data interpretation, it likely led to the exclusion of valuable research in other languages, which could have offered broader insights into the use of hyaluronic acid (HA) and ozone in dentistry [49,50].

Second, we limited our database search to PubMed, Google Scholar, and Ovid. While these are widely used, omitting other specialized databases, like Cochrane or Embase, may have restricted the scope of our review, potentially missing relevant studies from different regions or fields.

Additionally, by focusing only on human-controlled trials, we may have overlooked early-stage research, such as animal or in vitro studies, which could provide important preliminary data. There is also a risk of publication bias, as studies with positive results are more likely to be published, potentially skewing our findings towards the benefits of HA and ozone therapy.

Another challenge was the variability across the studies we reviewed. Differences in product formulations, application methods, and clinical evaluations made it difficult to draw consistent conclusions or recommend a standard treatment approach.

Finally, because our review was initiated in 2015, we may not fully capture the latest developments in HA and ozone therapy, especially given how quickly dental technologies and treatments are evolving.

## 5. Conclusions

Currently, HA and OT find extensive application across various medical disciplines, showcasing notable potential in dentistry, especially in managing both acute and chronic inflammatory conditions. An in-depth review of 28 clinical studies has illuminated HA’s beneficial impact on tissue repair and wound healing. This insight suggests that HA-OT topical application could be instrumental not only in the recovery phase following dental surgeries but also in addressing conditions such as gingivitis and periodontitis. Patients suffering from these dental ailments could see a marked enhancement in their overall quality of life thanks to the therapeutic properties of HA. Given these encouraging findings, it is prudent to pursue further investigations through laboratory research and more comprehensive, large-scale randomized controlled trials. Such endeavors are essential to validate HA-OT’s effectiveness fully and potentially expand its use in dental care practices.

## Figures and Tables

**Table 1:** Summary of study.

Criteria	Inclusion	Exclusion
Language	Studies published in English	Studies published in other languages
Study Design	Human-controlled trials (randomized controlled trials, clinal trails)	Literature reviews, technical notes, letters to editors, instructional courses
Population	Human subjects with dental conditions	Animal studies, in vitro research
Intervention	Use of hyaluronic acid (HA) and ozone in treating gingivitis, ulcers, wounds, gingival recession	Studies not involving HA or ozone in dental treatment
Outcome Measures	Histological or clinical evaluations of the impact of hyaluronic acid in dental disease contexts	Studies without clinical or histological evaluations
Publication Type	Primary research articles	Non-primary research (e.g., opinion pieces, conference abstracts)
Date Restrictions	No restriction on publication year	None
Study Topic	Focus on HA and ozone’s effectiveness in oral health treatments	Focus on unrelated medical conditions or treatments

**Table 2:** Explication of PICO.

PICO Component	
Participants	Healthy participants with no restrictions on age and sex with gingival recession, periodontitis, oral ulcers, surgery wounds
Intervention	Application of HA combined with OT in conjunction with surgical procedures
Comparison	The same surgical procedures without HA and OT or substitute
Outcomes	Pathology reduction

**Table 3:** Main studies included in this review (y: years; PBI: papillary bleeding index; HA; hyaluronic acid; API: approximal plaque index; DMF-T: decayed–missing–filled teeth; GI: gingival index; SFFR: sulcus fluid flow rate; BOP: bleeding on probing; PAL: probing attachment level; CAL: clinical attachment level; PD: probing depth; SRP: scaling root planing; MPO: myeloperoxidase; GFC: crevicular fluid volume; CHX: chlorhexidine; EHA: esterified low-molecular-weight HA; BnzHCL: benzydamine HCL; RD: recession deep).

Authors	Mean Age	Ha Group	Control Group	Type of Treatment	Parameters Evaluated	Clinical Evidence
Jentsch [2]	50 male (17 ± 39 y)	25 with use of HA-OT	25 with placebo	Gel on gingivitis.	The study evaluated oral health through clinical indices (approximal plaque index, Turesky plaque index, papillary bleeding index) and crevicular fluid markers (peroxidase, lysozyme) initially and after 4, 7, 14, and 21 days.	The test group exhibited notable enhancements in plaque indices from day 4 and in PBI from day 7, outperforming the placebo group.
Pistorius [16]	60 mixed (32 ± 41 y)	40 with use of HA-OT	20 with reduced use of HA	Spray on gingivitis.	Clinical measurements including DMF-T index, API, sulcus bleeding index, PBI, and gingival crevicular fluid were recorded at the start, then after 3 and 7 days.	Clinical parameters were assessed initially, and then at 3 and 7 days. The HA group saw decreases in sulcus bleeding index at both time points, with significant drops in PBI values and gingival crevicular fluid.
Badge [18]	21 mixed (22 ± 34 y)	11 with use of HA-OT	10 with placebo	Gel in periodontal pocket.	A gingival biopsy for histopathological and immunohistochemical analysis, focusing on Ki-67 expression and inflammatory infiltrate evaluation, was conducted 30 days post-treatment.	Treatment with HA gel notably decreased the proliferation index of gingival epithelium and fibroblast cells.
Sahayata [17]	105 mixed	50 with use of HA-OT	50 with reduced use of HA and short follow-up	Gel in periodontal pocket.	Clinical parameters (API, GI, PBI) were assessed at 1, 2, and 4 weeks from baseline; microbiological parameters were checked at 4 weeks.	Significant improvements in GI and PBI were observed in the test group compared to others. At 4 weeks, all treatment groups saw a significant decrease in anaerobic Gram-negative bacilli and an increase in Gram-positive coccoid cells from baseline.
Xu [19]	20 mixed (48 ± 64 y)	10 with use of HA-OT	20 with placebo	Gel in periodontal pocket.	SFFR and sulcus bleeding index were measured initially and weekly up to 12 weeks; probing depth and clinical attachment level were checked at the start and at 6 and 12 weeks. Dentists collected subgingival plaque samples to identify specific bacteria at baseline and at 6 and 12 weeks.	This study showed an improvement of all clinical variables in both groups. There are no clinical and microbiological differences between test and control sites.
Johannsen [20]	11 mixed (23 ± 56 y)	10 with use of HA-OT	11 with use of placebo	Spray in periodontal pocket.	SFFR and sulcus bleeding index were measured initially and weekly up to 12 weeks; probing depth and clinical attachment level were checked at the start and at 6 and 12 weeks. Dentists collected subgingival plaque samples to identify specific bacteria at baseline and at 6 and 12 weeks.	There are no clinical and microbiological differences between test and control sites.
Polepalle [21]	36 mixed (30 ± 65 y)	26 with use of HA-OT	10 with use of reduced HA and short follow-up	Gel in periodontal pocket.	Bleeding on probing (BOP), API, probing pocket depth (PPD), and clinical attachment level (CAL) were assessed at baseline, 1, 4, and 12 weeks. Colony-forming units (CFU) per mL were assessed at baseline, after treatment, and after 2 weeks.	There was a significant reduction in BOP, API, PPD, and CAL in the test sites than control group. In the test sites there was also a significant reduction of CFUs.
Gontiya [22]	26 mixed (25 ± 55 y)	20 with use of HA-OT	6 with use of placebo	Gel on gingivitis.	Clinical parameters GI, PBI, PPD, and relative attachment level (RAL) evaluated at baseline (day 0) and weeks 4, 6, and 12.	The test sites showed statistically significant improvement in GI and PBI at 6 and 2 weeks compared to control sites.
Rajan [23]	Not specified	33 with use of HA-OT	Not specified	Gel on gingivitis.	The clinical parameters evaluated: GI, API, BOP, PPD, CAL at three appointments: before SRP, 4 weeks, and 12 weeks after SRP.	The test sites showed statistically significant improvement in GI and PBI at 6 and 2 weeks compared to control sites.
Pilloni [24]	19 mixed (15 ± 41 y)	15 with use of HA-OT	4 with use of placebo	Gel and spray in mild chronic periodontitis.	These clinical parameters were evaluated before treatment and repeated at 14 and 21 days: API, BOP, GI, probing attachment level (PAL).	HA gel treatment was more effective, reducing BOP by 92.7% and GI by 96.5%, compared to 75.8% and 79.0% in controls. Periodontitis reduction was significantly greater in the HA-treated area.
Eick [25]	42 mixed (41 ± 72 y)	17 with use of HA-OT	17 with use of placebo	Gel and spray in mild chronic periodontitis.	PD and CAL measurements were taken at the start, 3 months, and 6 months, with subgingival plaque and sulcus fluid samples collected for analysis.	The test sites showed statistically significant improvement in GI and PBI at 6 and 2 weeks compared to control sites.
Chauhan [26]	60 mixed (30 ± 65 y)	30 with use of HA-OT	30 with use of reduced HA	Gel and spray in mild chronic periodontitis.	PD and CAL measurements were taken at the start, 3 months, and 6 months, with subgingival plaque and sulcus fluid samples collected for analysis.	At 3 months, change in PPD and CAL was greater in the test group than the control group, but the difference was non-significant.
Engstrum [27]	15 mixed (23 ± 54 y)	8 with use of HA-OT	7 with use of placebo	Not specified.	PD and CAL measurements were taken at the start, 3 months, and 6 months, with subgingival plaque and sulcus fluid samples collected for analysis.	After 12 months, the test and control groups in surgery showed a bone height difference under 1 mm, visible only in radiographs. Both groups experienced bone height reduction post-scaling. Probing depth decreased as anticipated following surgery and SRP.
Briguglio [7]	15 mixed (23 ± 54 y)	8 with use of HA-OT	7 with use of placebo	Not specified.	PD and CAL measurements were taken at the start, 3 months, and 6 months, with subgingival plaque and sulcus fluid samples collected for analysis.	The use of hyaluronic acid in treating infrabony defects provided additional advantages, including improved clinical attachment levels, reduced probing depths, and enhanced predictability, compared to traditional open flap debridement methods.
Bevilacqua [28]	24 mixed (+-51 y)	11 with use of HA-OT	13 with use of placebo	Gel in moderate–severe chronic periodontitis.	Clinical variables assessed included API, BOP, CAL, PPD, calprotectin, MPO, and GCF volume on days 45 and 90. Calprotectin, MPO, and GCF quantities were measured at test and control sites on days 7 and 45.	At baseline and 45 days, the HA group showed a significant decrease in probing depth and BOP compared to the control group. Both groups experienced a notable reduction in calprotectin and myeloperoxidase per sample after 1 week, followed by an increase at 45 days.
Karim [29]	14 mixed (23 ± 34 y)	7 with use of HA-OT	7 with reduced use of HA	Gel in chronic periodontitis.	BOP, API, PPD, and CAL were assessed at baseline, 1, 4, and 12 weeks. CFU per mL was assessed at baseline, after SRP, and after 2 weeks.	The test sites showed significant improvements in BOP, API, PPD, and CAL compared to the control group, alongside a notable decrease in CFUs.
Araujo Nobre [30]	30 mixed (58.4 ± y)	15 with use of HA-OT	15 with use of CHX	Management of the implant platform and healing screw at implant uncovering with gel.	The clinical parameters evaluated: modified plaque index (mPlI), modified bleeding index (mBI), PPD in mL, suppuration (Sup), clinical implant mobility (mob). Both groups were followed up for 6 months, and the clinical observations were performed on day 10 and at 2, 4, and 6 months post-surgery.	HA and CHX effectively supported peri-implant health. The HA group had significantly better modified bleeding index at the second check. At 6 months, CHX showed potentially superior outcomes in modified plaque and bleeding indices.
Galli [31]	8 mixed (36 ± 67 y)	4 with use of HA-OT	Not specified	Post-implant wound management with gel.	The PPDs, gingival recession, and CAL were evaluated before treatment and after 1 year.	After 1 year the following results were found: PPD reduction, gingival recession increase, and CAL gain.
Ballini [32]	19 mixed (43.8 ± y)	19 with use of EHA-OT	Not specified	Post-implant wound management with gel.	The PPDs, gingival recession, and CAL were evaluated before treatment and after 1 year.	Clinical results showed a mean gain of CAL (gCAL) of 2.6 mm at the treated sites, confirmed by radiographic evaluation.
Koray [33]	34 mixed (23 ± y)	34 with use of HA-OT	34 with use of BnzHCl	Management of bilateral extraction of the lower octaves with HA gel or BnzHCL spray.	Swelling was measured with a tape and trismus by the maximum interincisal opening. Evaluations occurred on the surgery day and 2 and 7 days post-surgery.	The patients with HA gel experienced statistically significant results for the swelling and trismus values compared to those with the BnzHCl spray.
Romeo [34]	49 mixed (45.5 ± y)	31 with use of HA-OT	18 with use of placebo	Management of excisional biopsy with HA gel.	The lesion area was measured after surgery (T0) and after 7 days (T1). A percentage healing index (PHI) was calculated, indicating healing extension in 7 days.	Not specified.
Kumar [21]	Not specified	1 with use of HA-OT	Not specified	Gel on gingival recession.	RD, PPD, and CAL were tracked at baseline and then at 1, 3, 6, 12, and 24 weeks post-surgery.	Despite the lack of statistical significance, the experimental group’s root coverage was observed to be more clinically stable than that of the control group at 24 weeks.
Lee [35]	50 mixed (40 y)	33 with use of HA-OT	17 with placebo	Gel on oral ulcers in Behçet’s disease.	Subjective assessment: number of ulcers, healing period and VAS; objective assessment: number and maximal size of ulcers.	Ulcer inspection revealed a 57.6% reduction in numbers and a 78.8% decrease in area among patients. Post-treatment, significant improvements were seen in swelling and local heat.
Nolan [6]	106 mixed (37 y)	60 with use of HA-OT	56 with use of placebo or reduced level of HA	Gel on oral ulcers.	Average ulcer count, ulcer history over 7 days, patients experiencing ulcers in this period, and treatment assessment scores ranging from very good to not recorded.	Both groups noted quick discomfort relief from ulcers, lasting around 30 min before reverting towards initial levels. Ulcer counts slightly dropped over 7 days, regardless of treatment. By day 5, the HA group reported significantly fewer ulcers compared to the placebo group. Despite new ulcers appearing in both groups during the study, the HA group saw a notably lower incidence of new ulcers by day 4.
Lopez [36]	1 male (32 y)	1 with use of HA-OT	Not present	Application of HA gel in intracrestal sinus lift.	The filling volume obtained was measured with a comparative software program and using an ellissoid formula. This technique allows the surgery to be performed in a way that is both minimally traumatic and invasive, fully careful of the membrane, and represents a viable alternative to those surgical techniques for crestal sinus lift currently in use.	Not specified.
Schwartz [37]	26 mixed (45 ± y)	26 with use of HA-OT	Not present	Application of HA gel and bone graft in lateral sinus lift.	All 32 sinus lifts succeeded, with cone beam scans showing bone height increasing from 2.84 mm pre-treatment to 15.2 mm post-treatment.	This study confirmed the hypothesis that new bone formation is graft dependent alone or in combination with other materials.
Weindl [8]	45 mixed (23 ± 45 y)	25 with use of HA-OT	20 with use of placebo	Treatment of gingival recession with use of HA gel.	Recession depths in the first, third, and sixth month were 1.82 ± 0.442, 1.31 ± 0.47 mm, and 0.91 ± 0.29, respectively, which showed a significant reduction.	Within the limitations of the present study, the data obtained by periodic assessment of the clinical parameters indicate the use of amnion membrane and hyaluronic acid, and proper technique may thus be the panacea for root coverage procedures.
Gorski [38]	24 mixed (34 ± y)	24 with use of HA-OT	Not applicable	Use of HA gel in the treatment of multiple gingival recession using the modified coronally advanced tunnel technique (MCAT) combined with subepithelial connective tissue graft (SCTG), with or without cross-linked hyaluronic acid (HA).	No significant improvement in root coverage was observed because of adding HA. After 6 months, mean root coverage (MRC) was 85% for SCTG + HA group and 83% for SCTG group (*p* = 0.9819). Complete root coverage (CRC) was observed in 91% (test) and 93% (control) of the cases (*p* = 0.9001).	Both treatments were similarly effective in treating multiple GRs and led to comparable improvements in clinical parameters. However, application of HA improved the appearance of soft tissue texture.

## Data Availability

The data from the present study can be obtained upon reasonable request from the corresponding author.
